# Supplementary material for: MetaRibo-Seq measures translation in microbiomes
Source: Nat Commun. 2020 Jun 29;11:3268. doi: 10.1038/s41467-020-17081-z (PMC7324362; doi:10.1038/s41467-020-17081-z)
Supplement: Supplementary file 10 — Supplementary Data 7 [file 41467_2020_17081_MOESM10_ESM.zip › File2/Confidence_High_Taxonomy/138801_out.krona.html]

Javascript must be enabled to view this page.

members
magnitude
magnitudeUnassigned
count
unassigned
taxon
rank

138801\_out

2

2
2
superkingdom

phylum
1239
2

class
186801
2

order
186802
2

1
31979
family

genus
1485
1

species
1

SRS064276\_contig\_number\_43133
2293016

family
1
186803

genus
1
841

1

SRS018836\_contig\_number\_contig-100\_112.113
360807
species
